# Rethinking Radiology: An Active Learning Curriculum for Head Computed Tomography Interpretation

**DOI:** 10.5811/westjem.2021.10.53665

**Published:** 2022-01-01

**Authors:** Leonardo Aliaga, Samuel Owen Clarke

**Affiliations:** University of California – Davis, Department of Emergency Medicine, Sacramento, California

## Abstract

**Introduction:**

Head computed tomography (CT) interpretation is a vital skill for emergency physicians. Existing literature shows poor concordance between emergency physicians and radiologists in head CT interpretation. Prior studies have used passive learning methods to address this knowledge gap. We created an active learning curriculum for teaching head CT interpretation to emergency medicine (EM) residents and compared its effectiveness to a passive learning strategy.

**Methods:**

We conducted a prospective, randomized controlled study of EM residents at a single institution. Three educational sessions were delivered over a three-month period via video conference. The active learning cohort (ALC) scrolled through head CT teaching cases we designed on Pascbin, a web-based radiology picture archiving and communication system. The passive learning cohort (PLC) watched instructional videos that scrolled through the same cases. Both cohorts were given equal time to review the cases and ask an instructor questions. Residents took pre-intervention and post-intervention tests on head CT interpretation. We analyzed scores using paired and unpaired t-tests.

**Results:**

Forty-two residents took the pre-intervention test. Mean pre- and post-test scores for the ALC were 43.8% and 59.0% (P <0.001), and for the PLC were 41.7% and 45.3% (P = 0.29). The difference in ALC and PLC post-test scores was statistically significant (P = 0.009) with a large effect size (Cohen’s d = 1.34).

**Conclusion:**

Our active learning head CT curriculum using Pacsbin showed superior learning outcomes when compared to a passive learning strategy and required no additional time or resources. This intervention offers a more effective and learner-centric method for implementing radiology curricula in EM residency programs.

## INTRODUCTION

Emergency physicians (EPs) must be able to identify life-threatening and time-sensitive findings on head computed tomography (CT) that require immediate action, often before a radiologist’s report is available.^1^,^2^ Learning to accurately interpret head CTs requires detailed instruction and repeated exposure to both normal studies and diverse pathologic findings, elements that are difficult to achieve in a time-restricted setting such as emergency medicine (EM) residency didactic conference. Perhaps unsurprisingly, head CT interpretation concordance between EPs and radiologists has been shown to be poor across a variety of practice settings.^1^–^7^

Prior studies addressed this knowledge gap using one-time didactic lectures or instructional videos.^8^–^12^ However, demonstrating CT findings on single images poorly represents the cognitive work of identifying these findings in clinical practice.^13^ Even if a lecturer “scrolls” through a CT, learners are unable to actively engage with the images. While we know that passive learning methods lead to poorer retention,^14^ active learning curricula for radiographic interpretation have remained elusive. Despite a body of evidence supporting the benefits of active learning,^15^–^22^ time and resource barriers exist to implementing these methods into residency didactic curricula.^18^–^24^

Pacsbin (Orion Medical Technologies, Baltimore, MD) is a web-based radiology picture archiving and communication system (PACS) that provides learners with a familiar platform to scroll through CTs, simulating the way they engage with imaging studies in clinical practice and providing a potential vehicle for active learning (www.pacsbin.com).^25^–^29^ While practice cases on various PACS platforms have supplemented existing curricula,^27^–^29^ no prior study has used this technology to directly compare active and passive learning strategies.

To evaluate this approach, we created an active learning-based curriculum using Pacsbin for teaching head CT interpretation to EM residents. Our objective was to compare the effectiveness of this active learning approach to a passive learning strategy within our didactic conference while maintaining resource neutrality in terms of time and access to instruction. We hypothesized that EM residents who learned head CT interpretation using our active learning curriculum would demonstrate greater diagnostic accuracy on a head CT interpretation test.

## METHODS

### Study Population and Design

This study was conducted at the University of California, Davis EM residency program and approved by our institutional review board. We used a convenience sample of first-, second-, and third-year residents at our institution. As this was a pilot study, we did not perform an *a priori* power calculation. After consent, residents took a pre-test of head CT interpretation and were subsequently randomized to an active learning cohort (ALC) or passive learning cohort (PLC). Three educational sessions (on intracranial hemorrhage, acute ischemic stroke, and increased intracranial pressure) were delivered monthly over a three-month period via Zoom conference (Zoom Video Communications, Inc., San Jose, CA). The ALC convened in a virtual breakout room where residents accessed head CT teaching cases on Pascbin using their individual computers (cases in Supplement). Pacsbin simulates a radiology PACS, allowing learners to scroll through CTs (including axial/coronal/sagittal views), annotate images, adjust brightness and contrast, and access built-in links to instructional diagrams. Residents scrolled through head CTs guided by teaching points built into each case. After finishing the cases the ALC had a 10-minute, live question-and-answer (Q&A) session led by one of the investigators.

The PLC watched pre-recorded instructional videos in a live, synchronous fashion via video conference which was immediately followed by a 10-minute, live Q&A session. These videos scrolled through the same cases and explained the same teaching points the ALC received through Pacsbin. We controlled the length of these educational sessions using virtual breakout rooms with a pre-set time limit of 60 minutes. Residents took a post-test one month after the last session using the same questions on the pre-test. We tested the data for normality and analyzed pre- and post-test scores using paired and unpaired t-tests.

### Head Computed Tomography Test and Active Learning Cases

We created a head CT interpretation test on Pacsbin and pilot tested it with three EM education and simulation faculty to collect content and response process validity evidence for the instrument. All faculty agreed the test cases represented critical knowledge and skills needed in EM and noted there was an appropriate range of difficulty. Faculty agreed the image quality was essentially identical to what we would encounter on our institution’s radiology PACS. Feedback from faculty was used to revise the test. We reviewed and modified answer choices across all the questions to reduce potential construct-irrelevant variance from learners inferring correct/incorrect choices based on where they appeared.

The test included cases with obvious pathologic findings as positive controls (e.g., classic “star” pattern of acute subarachnoid hemorrhage filling the basal cisterns, large acute subdural hematoma) and normal studies as negative controls. We built active learning modules on Pacsbin. Each module consisted of six to eight cases illustrating critical findings relevant to EM practice and normal comparisons. The modules guided the learner to incorporate predefined heuristics for identifying critical findings. The videos watched by the PLC presented the same heuristics and cases (test, modules, and videos in Supplement). The primary author completed four years of neurosurgery residency before switching to EM and used his expertise in head CT interpretation to develop the learning modules and heuristics.

## RESULTS

Forty-two residents took the pre-test. Twelve residents in the ALC and eight residents in the PLC completed all three educational sessions and took the post-test. Test score distributions passed the Shapiro-Wilk normality test. Mean pre-test scores and 95% confidence intervals (CI) were as follows: for the ALC 43.8% (CI: 38.0–49.5), and for the PLC 41.7% (CI: 36.5–46.8) (P = 0.62). Mean post-test scores and 95% CI were as follows: for the ALC 59.0% (CI: 53.3–64.8), and for the PLC 45.3% (CI: 38.2–52.5) (P = 0.009) (Figure). The score increase for the ALC was statistically significant using a paired t-test (*P* <0.001); however, it was not for the PLC (*P* = 0.29). The effect size was large when comparing the ALC and PLC post-test scores (Cohen’s d = 1.34).

## DISCUSSION

While EPs do not need the same level of mastery in head CT interpretation as radiologists, they must be able to identify critical and time-sensitive findings, often before a radiologist’s report is available.^1^,^2^ This is particularly true in practice settings that do not have attending radiologists in house at all times.^10^,^30^,^31^ Nonetheless, the skill of head CT interpretation exists in a border region of knowledge domains between clinical specialties. As educators, this forces us to consider the complex issue that teaching one topic to sufficient depth can come at the expense of time for other topics in residency education. In this study, we designed an evidence-based and learner-centered solution for teaching head CT interpretation and found this was achievable within the time and resource constraints of our residency’s didactic conference curriculum.

Active learning is rooted in constructivist learning theory and posits that learners build knowledge frameworks through active engagement with learning material.^32^ Despite extensive evidence supporting active learning approaches, passive learning remains the dominant modality in most educational settings.^14^,^19^,^21^,^22^ Avoidance of active learning may be related to the perception that it requires extra time or resources; however, our intervention fit into an existing residency didactic conference schedule without requiring extra time either during or outside the session.

Our novel curriculum created active engagement by making learners scroll through head CT images themselves, setting the conditions for active learning and accurately reproducing the cognitive work used to identify these findings in clinical practice. This intervention embedded the didactic content into a Pascbin and incorporated all the skills needed to correctly interpret a non-contrast head CT (e.g., manipulating window presets, brightness and contrast, and identifying pathologic findings in relation to key anatomic structures). This forced learners to interact with the didactic content in a way that is lost with lectures or videos. This approach to teaching head CT interpretation has not been previously described in the literature and represents an important step forward from the historical reliance on passive learning strategies to address this key content area.

We designed this study to specifically isolate the influence of passive vs active engagement with the learning material. Both the ALC and PLC were exposed to identical cases, embedded prompts, and questions. The two groups received identical amounts of time to review the material using Zoom breakout rooms to control length of exposure, and both received the same amount of time to ask clarifying questions. The crucial difference between the two groups was how they engaged with the learning material. The PLC watched videos where an instructor scrolled through cases whereas the ALC had to scroll through cases themselves. Given that all other learning conditions were controlled for, we hypothesize that making residents in the ALC search for and identify key findings on their own may have facilitated deeper knowledge encoding and greater improvement in diagnostic accuracy.

It is notable that the PLC did not significantly improve despite receiving the same content, teaching points, and heuristics. The videos watched by the PLC were made to be engaging, clear, and easy to follow. It is possible the videos’ cognitive fluency produced an illusion of learning and robbed the viewers of effortful learning, leading to poorer retention.^16^,^33^–^35^ In contrast, the ALC had to scroll through images and search for findings, which likely contributed to some degree of effortful learning. We realize that the three 60-minute Pacsbin sessions given to the ALC were insufficient to ensure complete understanding of all this content, despite showing improved performance compared to the PLC. However, this work serves as a proof of concept and a potential springboard for spaced repetition. After residents complete the initial modules, single cases can be delivered synchronously or asynchronously and completed in a shorter time frame. We suspect these subsequent cases might serve as booster inoculations, strengthening knowledge encoding and potentially improving scores on future tests.

## LIMITATIONS

This pilot study is not without limitations. We collected content and response process validity evidence for our head CT interpretation test; however, this evidence relied on expert (i.e., attending-level) opinion and might have been strengthened by incorporating junior learners. The intervention was conducted without an *a priori* power calculation and used a convenience sample of residents at a single EM residency program, limiting its generalizability. We used pre-recorded instructional videos as our passive learning control, which differ from traditional lectures and limit our results’ generalizability. However, the videos allowed us to standardize the control intervention while providing some resemblance to lectures by being shown in live, synchronous fashion followed by a Q&A session. Our study also suffered from attrition, with 22 residents missing one or more educational sessions due to schedule conflicts. We nonetheless found a large effect size despite a relatively small sample, highlighting the potential impact of our intervention.

## CONCLUSION

Our active learning head CT curriculum using Pascbin led to greater diagnostic accuracy when compared to a typical passive learning strategy. We achieved this superior outcome while maintaining resource neutrality in terms of time and access to instruction. We believe this study adds to the landscape of active learning literature by demonstrating an effective way to strengthen radiology curricula in EM residency programs.

## Supplementary Information



## Figures and Tables

**Figure:**
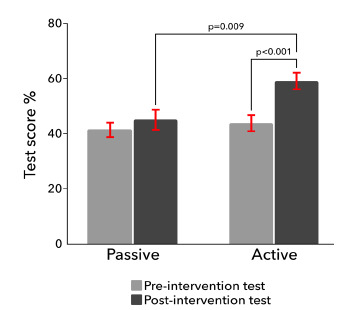
Pre- and post-intervention test scores for passive and active learning cohorts.
