# Supplementary material for: Rethinking Radiology: An Active Learning Curriculum for Head Computed Tomography Interpretation
Source: West J Emerg Med. 2022 Jan 1;23(1):47–51. doi: 10.5811/westjem.2021.10.53665 (PMC8782134; doi:10.5811/westjem.2021.10.53665)
Supplement: Supplementary file 1 [file wjem-23-47-s001.docx]

Rethinking Radiology: An Active Learning Curriculum for Head Computed Tomography Interpretation

**SUPPLEMENTAL MATERIAL**

**Head CT Interpretation Test**

A single test was developed and used for both the pre- and post-intervention assessment.  The test contained 24 questions on 20 individual head CT cases. Residents accessed the head CT images on Pacsbin through weblinks associated with each question. Once the pre-test was administered the weblinks were disabled and residents were unable to access the radiology cases and questions until the post-test was administered.

<https://forms.gle/B84VgGRvstGVahJA8>

**Active Learning Modules**

We created three modules, each focusing on one topic. The modules used head CT cases we uploaded to Pacsbin. Patient information was removed from all cases. Residents completed each of these modules once, virtually and synchronously, during didactic conference time and did not have access to them afterward.

Identifying acute intracranial hemorrhage: [Head CT Heuristics – 1](https://drive.google.com/file/d/1m5dMjCmZPY43yRpPQjki5_z68UnUfLer/view?usp=sharing)

Identifying acute ischemic stroke: [Head CT Heuristics - 2](https://drive.google.com/file/d/1pYccvnCG_gF5-p47q1L5enFYim-m3E0l/view?usp=sharing)

Identifying increased intracranial pressure: [Head CT Heuristics – 3](https://drive.google.com/file/d/1nOUOkNfb5g5b9GY6OsR2BYP5xNPsUD97/view?usp=sharing)

**Passive Learning Videos**

We created three instructional videos that used the exact same head CT cases featured in each of the active learning modules. The videos were made to be engaging as well as making sure to highlight the exact same learning points in the active learning modules. Residents viewed each of these videos once, synchronously during didactic conference time and did not have access to them afterward.

Identifying acute intracranial hemorrhage: <https://youtu.be/W__Q5iC2vA4>

Identifying acute ischemic stroke: <https://youtu.be/AOTDPmFXEwk>

Identifying increased intracranial pressure: <https://youtu.be/2NDGhcRgpiY>
